# A Tangle of Genomic Aberrations Drives Multiple Myeloma and Correlates with Clinical Aggressiveness of the Disease: A Comprehensive Review from a Biological Perspective to Clinical Trial Results

**DOI:** 10.3390/genes11121453

**Published:** 2020-12-03

**Authors:** Mariarosaria Sessa, Francesco Cavazzini, Maurizio Cavallari, Gian Matteo Rigolin, Antonio Cuneo

**Affiliations:** Hematology Section, Department of Medical Sciences, Azienda Ospedaliero-Universitaria, Arcispedale S.Anna, University of Ferrara, 44121 Ferrara, Italy; cvzfnc@unife.it (F.C.); cvlmrz@unife.it (M.C.); rglgmt@unife.it (G.M.R.); cut@unife.it (A.C.)

**Keywords:** multiple myeloma, genomic aberrations, clinical trials

## Abstract

Multiple myeloma (MM) is a genetically heterogeneous disease, in which the process of tumorigenesis begins and progresses through the appearance and accumulation of a tangle of genomic aberrations. Several are the mechanisms of DNA damage in MM, varying from single nucleotide substitutions to complex genomic events. The timing of appearance of aberrations is well studied due to the natural history of the disease, that usually progress from pre-malignant to malignant phase. Different kinds of aberrations carry different prognostic significance and have been associated with drug resistance in some studies. Certain genetic events are well known to be associated with prognosis and are incorporated in risk evaluation in MM at diagnosis in the revised International Scoring System (R-ISS). The significance of some other aberrations needs to be further explained. Since now, few phase 3 randomized trials included analysis on patient’s outcomes according to genetic risk, and further studies are needed to obtain useful data to stratify the choice of initial and subsequent treatment in MM.

## 1. Introduction

Multiple myeloma (MM) is a plasma cell disorder, accounting in term of prevalence for about 10% of hematological malignancies. MM is a genetically heterogenous disease arising from the accumulation of genomic events over the years [1]. The model of clonal progression from a pre-malignant stage of monoclonal gammopathy of uncertain significance (MGUS) through an asymptomatic stage named smoldering multiple myeloma (SMM) to MM and plasma cell leukemia (PCL) is well studied [2,3,4]. The accumulation of mutations and chromosome abnormalities over the time [5] favor us in considering MM a model of cancer genesis and evolution.

The process of tumor genesis and progression is triggered by several recurrent events at the genome and chromosome level. Some events typically occur at diagnosis, whereas others could be detected in most patients in late stages of the disease [6]. Furthermore, the concurrence of several and genetically heterogeneous subclones within MM cells—in a context of selection pressure—complicates the interpretation of the genetic of the tumor for intrinsic spatial [7,8] and population heterogeneity [9,10,11]. In recent years, whole-exome (WES) and whole-genome sequencing (WGS) studies highlighted the complex genomic aberrations underlying the pathogenesis of the disease [12,13,14,15].

This review aims to present the tangle of genomic events that drive plasma cell disorders, particularly MM, and to focus on their clinical significance.

## 2. Landscape of Genomic Damage in MM

### 2.1. Timing of Genome Aberrations in MM

MM has been studied by systematic analysis of target sequencing, WES, and single nucleotide polymorphisms (SNP) array, to identify DNA aberrations recurring at high and intermediate frequencies, with the aim to appreciate pathogenetic dysregulated pathways and potential therapy targets. The analyses revealed in MM a somatic mutation rate of 1.6 mutations per MB [11,13,14,16,17,18,19,20,21,22].

Plasma cell disorders are genetically heterogeneous diseases and their clonal progression occurs in sequential phases [11]. Hyperdiploid (HD) events are characterized by the acquisition of chromosome trisomy. Frequently, trisomy occurs in chromosome 3, 5, 7, 9, 11, 15, 19, 21 [19,23]. HD is the main event identified in early stage MM [11]. Non-HD events occurring in early stage MM pathogenesis are characterized by the well-known translocations typically affecting the genes encoding immunoglobulin (Ig) heavy chains (IGH), mainly t(4;14), t(6;14), t(11;14), t(14;16), and t(14;20) [19]. HD MM carries a lower prevalence of primary translocations, as opposed to non-HD MM. Due to their different pathogenesis, they are recognized as the two main genetic subtypes of MM. Secondary events, detected generally in later stages, are essential for tumor progression and consist of chromosomal translocations, copy-number variations (CNV) and single-nucleotide somatic variations that affect several signaling pathways, cell-cycle regulators, and DNA-damage repair mechanisms [19].

Aktas Samur recently described the chronology of copy number aberrations in 164 MGUS and 336 newly diagnosed MM. HD MM seems to originate from different events if compared with non-HD MM. In HD MM, CNVs are also clonal at the MGUS stage, ratifying that these are early events leading to transformation of normal plasma cell to pathologic MGUS cell but are not sufficient to generate MM proliferating cells. The proposed model of HD myeloma genesis and progression suggests the initial gain of at least two copy of chromosome 9, 15, and 19 also present in the MGUS stage; some patients then acquires trisomy of chromosome 6, or chromosome 18, or chromosome 21, while other patients lose chromosome 13 and/or gain 1q (about 20% of patients show no additional CNVs). In non-HD MM, IgH translocations are the earliest event, also identified in MGUS, and most patients do not accumulate other additional changes. In both groups, late catastrophic genomic events can occur, leading to complex deletions, irrespective of initial aberrations [24]. A mention must be referred to TP53 aberrations. Both mutations of the TP53 gene and deletions of chromosome 17p13 containing the TP53 gene are late events in MM history [9,25]. Deletion of 17p occurs with a frequency of about 9.5% in newly diagnosed symptomatic MM patients, rising up to 50% in primary plasma cell leukemia and to 75% in secondary plasma cell leukemia patients [19,26]. Similarly, TP53 mutations occur in 5–8% of newly diagnosed symptomatic MM patients, rising up to 25% in PCL [26,27,28,29,30]. TP53 mutations usually appears after or concurrently with deletion 17p13 [31]. Conversely, TP53 mutations are identified in one third of 17p deleted patients [27].

Damages arise in clone or sub-clone in MM. Early mutations in MM evolution are expected to be clonal. Mutations mostly occurring at the sub-clonal level arise later in MM progression. Nevertheless, several driver gene mutations can be found in MM at the sub-clonal level, underlining the heterogeneity of the cell population. For example, Bolli et al. identified sub-clonal mutations of the driver genes KRAS, NRAS, TP53. TP53 was sub-clonal in 26.7% and clonal in 11% of case. No significant differences were identified in prognosis when a mutation was identified at the clonal or sub-clonal level [5]. Data are lacking about clonal selection induced by treatment in MM. An analysis by Corre et al. evaluated 43 homogeneously treated MM patients for mutational profiles at the time of diagnosis and at relaps [32]. No specific or significant events in mutational status were found after treatment neither at relapse. Every patient seems to evolve differently, with chemoresistance and relapse driven by acquired mutations or selection of pre-existing subclones [32].

### 2.2. Mechanisms of DNA Damage in MM

**Figure 1:**
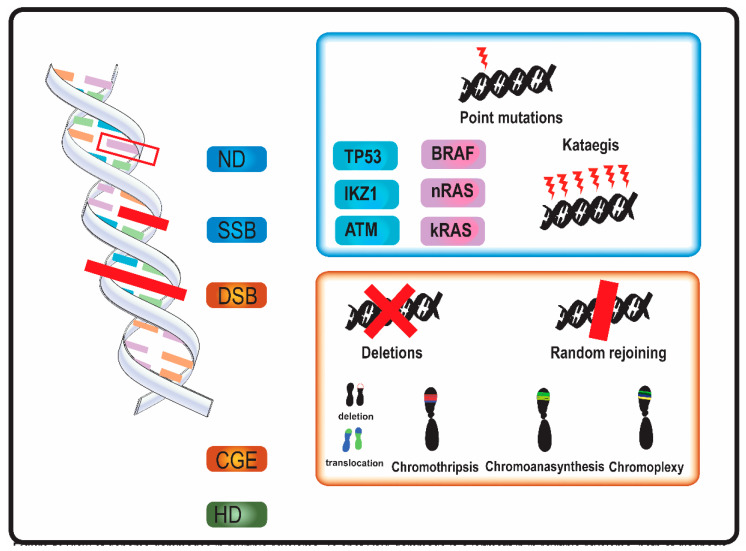
Overview of genome aberrations in Multiple Myeloma (MM). DNA damage is a leitmotif in in multiple myeloma, both at diagnosis and relapse. Nucleotide damages (ND) and single strand breaks (SSB) cause base substitutions that alter proteins and cell function. Frequent point mutations of clinical significance occur in genes involved in DNA integrity (in blue, e.g., TP53) and in cell cycle genes (in purple, e.g., kRAS). A DNA-level complex event named kataegis—a cluster of hundreds to thousands of point mutation in less than few megabases—was occasionally described in multiple myeloma. Double strand breaks (DSB) and complex genome events (CGE) widely alter genome and chromosome structure. Other than classical karyotype events (i.e., sub-chromosome deletions and translocations), in multiple myeloma were described chromothripsis, a one-step catastrophic event in which one or few chromosome were fragmented and randomly rejoined, with casual gain and losses of DNA (blue and red in figure, respectively); chromoanasynthesis a sub-chromosomal local cluster of duplications and triplications on one single chromosome (green clusters in figure); chromoplexy, a series of chained, complex inter- and intra-chromosome translocations, involving 1 to 8 chromosomes, with frequent loss of material at breakpoints. Alterations of ploidy and translocations are the 3rd category of genome abnormalities in MM, and hyperdiploidy (HD) is the main alteration in chromosome number.

In MM, genomic aberrations arise from various types of DNA damage (Figure 1). Nucleotide damage (ND) and single strand breaks (SSB) hesitate in single base substitutions. Double strand breaks (DSB) often alter chromosome structure.

Mutational events as kataegis and complex genome events (CGE) were described in MM [11,16,17,18,19,20,21,22]. Kataegis and, in general, “apolipoprotein B mRNA editing enzyme, catalytic polypeptide-like” (APOBEC) family-related signatures (a signature with C > T mutations in regions rich in 5-methyl-CpG and another one consisting C > T and C > G mutations in TCN trinucleotide repeats, which results from overactivation of cytidine deaminase activity) were observed in MM [5,11,19].

All the CGE described in MM, chromothripsis, chromoanasynthesis, and chromoplexy—also grouped together under the name of chromoanagenesis—arise from DSB [33]. SNP-microarray and WGS are able to identify these CGE also when localized genomic aberrations are not detected by conventional cytogenetic and Fluorescent in situ hybridization (FISH) analysis [17,34,35]. Genomics events bring a rapid genome variation and could also play a role in genome adaptive evolution [36].

Alterations of ploidy and translocations are frequent as a result of a disfunction in control mechanisms in late mitosis [37]. HD and translocations are the most common events historically described as driver events in MM.

Table 1 reports most frequent DNA damage events, as described in recent genomic studies.

#### 2.2.1. Single-Nucleotide Variants and Indels

WES identified recurrent somatic mutations in MM that drive pathogenesis. Three studies, including 203, 67, and 463 patients respectively, reported that the most frequently involved genes belong to the MAPK pathway, including KRAS, NRAS, and BRAF, mutated in about 40% of cases [10,11,38]. KRAS, NRAS, and BRAF were clonal in about 70% of the patients and sub-clonal in 30% in a cohort f 203 mixed patients, but treatment can select stronger clones, so additional studies are needed to define early from late events [10]. NF-KB is the second most affected pathway in MM. Mutations in this pathway can be found in about 20% of cases, in genes such as TRAF3, CYL, LTB, IKBKB, BIRC2, BIRC3, CARD11, and TRAF3IP1 [10,38,49]. About 15% of MM patients harbored mutations in genes involved in the DNA-repair pathway (TP53, ATM, and ATR) [38]. Recently, TP53 gene mutations were specifically studied with WES in 54 MM samples, due to the well-known poor prognostic role; 7/54 harbored TP53 mutation, 5/54 TP53 mutation together with del(17p). Among them, only 1 patient had TP53 mutation at diagnosis [39]. In addition, tumor suppressor genes DIS3 and FAM46C are frequently mutated in MM together with genes involved in B-cell lineage differentiation as PRDM1, IRF4, LTB, and SP140 [9,10,38].

Recent studies recognized an association between CRBN expression levels and response to IMIDs in MM. Therefore, SNVs in the CRBN gene have been investigated, but their role remains controversial due to the small number of patients [50,51].

#### 2.2.2. Copy Number Variations

Copy number variations (CNVs) can be primary, such as HD genome, or secondary events in MM, ranging from fragments to chromosomal arm deletions or amplifications [43,52,53].

CNVs can be located in focal regions, with high recurrence and contribution in MM pathogenesis. Recurrent and focal CNVs comprise amplifications and deletions and could be associated with prognostic significance [10,53].

Chromosome level copy number variation frequently occurs in newly diagnosed MM. Most frequent amplifications found with chromosome banding analysis or FISH are gain of 1q (31.3%), 6p (15.2%), and 11q (10.5%) [42] and loss of 16q (28.3%), 1p (24%), 14q (23.4%), 8p (22%), 6q (20.8%), 16p (14.7%), 12p (13%) [42]. Deletion of chromosome 17 p arm is found in about 10% of newly diagnosed MM, and up to 80% in later stages [40,41]. Mutations in TP53 gene coexist in 25–40% of patients with del(17p) [27,38]. The most frequent gains by SNP array analysis reported in a study by Walker were amp1q (36%), trisomy 15 (36.8%), 9 (35.9%), 19 (33.3%), 5 (33.3%), 3 (27.2%), 11 (24.6%), 7 (21%), 21 (12.3%), and X (8.7%); the most frequent deletions were 13q (59%), 14q (39%), 16q (35%), 6q (33%), 1p (30%), 8p (25%), 22 (18%), 12p (15%), 20 (12%), and 17p (7%) [43]. A recent analysis confirmed that del13 is the most frequent CNV (60%), followed by 1q gain (37%) and 14q deletion (32%). Deletion of chromosome 13 and 11 gain are early events in MM history [24].

HD is defined as a number of chromosomes ranging from 48 and 74. HD is a primary event in clonal progression and is identified in about 50% of cases of MGUS, SMM, and MM at diagnosis [19,24,44,45,46]. As a hypothesis, the gain of such a wide quantity of genetic material could be the result of a single catastrophic mitotic event [37]. In HD MM patients, the most frequent events are gains in chromosome 19 (95%), 15 (90%), and 9 (90%). More than 96% of patients carry gains of at least two of these three chromosomes. The other odd number chromosomes (5, 11, 3, 7, 21) follow in frequency. Moreover, Del13 is detected in 37% of HD MM patients [24]. HD cells can sometimes harbor coexisting MYC translocation [19,54].

#### 2.2.3. Chromosomal Translocations

Chromosomal translocations can occur early in MM pathogenesis, also being detected in the MGUS stage [55,56,57]. Translocations, frequently occurring during class-switch recombination, mostly affect the IGH locus 14q32.33 on chromosome 14, and result in upregulation of several oncogenes under control of IGH enhancer [58,59]. The most frequently recurring translocation is t(11;14), with 15–20% of prevalence. t(4;14), t(14;16), t(6;14), and t(14;20) occur in 15%, 5%, 1–2%, and 1% of multiple myeloma patients, respectively [19]. Partner genes can be mutated in 10–25% of cases, probably as a result of somatic hypermutation events [19].

Apart from these main translocations, chromosomal translocations involving MYC (and leading to its overexpression) can be found as secondary genomic events in 15–20% of patients with newly diagnosed MM, but only in 3–4% of patients with MGUS or SMM [19,47,48]. Translocations of MYC are identified in about 65% of MM with HD pattern MM [38]. The main partners of MYC translocations are the IGH genes (16.5%), IGL (16.5%), and IGK (6%), but also FAM46C (9.5%), FOXO3 (6%), and BMP6 (3.5%) [19]. These translocations are associated with the mechanism of kataegis [60].

#### 2.2.4. Mutation Clusters

Kataegis is a rare genome event occurring as a consequence of deregulated APOBEC activity on single-stranded DNA (ssDNA), leading to localized hypermutation around the breakpoint sites, before their reparation [13,61]. Cluster of hypermutation are usually several hundred base pair long [13,60,61]. The APOBEC family comprises cytidine-deaminase enzymes targeting ssDNA with high enrichment of C > G and C > T mutations during DNA replication (APOBEC signature), resulting in replicative asymmetry [60,62].

Bolli et al. first identified clusters of kataegis in MM samples [11]. In whole exome sequencing (WES) of 463 MM patients, kataegis was identified in sites of MYC translocation and in other sites. Furthermore, Walker and colleagues observed different complex signatures also associated with APOBEC family activity and eventually linked to deregulation of MAF and MAFB expression. APOBEC signatures were related to poor prognosis in patients enrolled in UK Myeloma XI study [19].

#### 2.2.5. Complex Genome Events

Chromothripsis is a CGE consisting in a major genome aberration, defined by the presence of massive rearrangements, clustered in localized chromosomal regions. Ten to hundreds of rearrangements accumulate in a rapid process generated by a single catastrophic event [63,64,65,66]. The classical hallmark of chromothripsis is the oscillation between two copy-number states [67]. Chromothripsis arises from two main mechanisms: the encapsulation of chromosomes in aberrant structures called micronuclei, with subsequent fragmentation and reassembly in a single chromatid, [65] and the fragmentation of DNA bridges formed between dicentric chromosomes during telomere crisis [68,69]. The PCAWG Consortium analyzed whole-genome sequences (WGS) of 2658 tumor samples including 38 cancer types searching for chromothripsis. The analysis revealed that chromothripsis is very common in cancer with a frequency greater than expected from previous studies, probably due to the presence of frequent additional aberrations which can mask the classical pattern [63]. In large MM patient sets, chromothripsis was detected at diagnosis with high-resolution single nucleotide polymorphism (SNP) arrays with a prevalence between 1.3% [20] and 10.0% [18] and was linked to poor prognosis in terms of progression free survival and overall survival [15,18,20]. In a recent low coverage WGS research, chromothripsis was identified in 24% of 752 newly diagnosed MM patients [15]. Chromosomes most frequently involved in MM were chromosome 1, 11, 12 [21], and 17 [18] even if other chromosomes were occasionally involved [16,17,18]. A recent analysis of WGS of MM patients revealed that chromothripsis can be a driver event in MM pathogenesis and mostly occur early in the history of the disease [17]. Furthermore, chromothripsis detection could also be important at relapse, as a next generations sequencing analysis showed a high prevalence of chromothripsis in resistant MM cell lines and in MM patients, and it was also correlated with resistance to bortezomib [21].

Chromoanasynthesis is another CGE arising from a single-step event during defective DNA replication. The block of template switching at the replicative fork site leads to a regional copy-number gain and to the insertion of short nucleotide sequences at the breakpoint junctions [36,70,71,72]. The underlining mechanism of incorporating fragments of DNA can be the same as in chromothripsis with defective DNA replication occurring in micronuclei. [73]. Chromoanasynthesis originates from errors in two DNA-repair mechanisms, fork stalling plus template switching, and microhomology-mediated break-induced replication, during endogenous or exogenous stress conditions [71,72,74]. Very few studies report chromoanasynthesis data for MM. Although it can be supposed to be a significant event in disease pathogenesis and progression, at this time, no conclusions can be done [16,22]. Recently, templated insertions were identified as the second most frequent complex event in 19% of 752 newly diagnosed MM patients [15].

Chromoplexy is a large aberration, first described in the prostate cancer genome, arising from a single catastrophic event [75,76]. It is defined by the presence of close chains of translocations involving many chromosomes (up to eight), with little or no copy number aberration. Chromoplexy raises from DNA double strand breaks that pair each other at breakpoint ends in a sequence of inter- and intra-chromosomal translocations [77,78]. The process could be triggered by physical clustering of breakpoint sites during DNA replication as in the micronuclei model [36,79]. In MM, chromoplexy was identified with WGS if 5 of 107 newly diagnosed patients, and involved more than 6 chromosomes [18]. A longitudinal WGS study reported chromoplexy in 3 of 67 patients, as a late event in MM history, with one patient showing at progression from asymptomatic to symptomatic MM and two patients at relapse [17]. The last low coverage WGS research reported chromoplexy in 11% of 752 newly diagnosed MM patients, confirming previous data [15].

## 3. Clinical Implications of Genome Aberrations in MM

Genetic events in MM can carry specific clinical significance, in terms of drug targeting, drug resistance, and prognosis.

### 3.1. Impact on Prognosis of Genome Aberrations and Clinical Trials Results

The most advanced results in stratifying MM patients are based on genomic lesions, derived by the Myeloma XI trial. A new prognostic model was defined, adding genomic events of relevant prognostic significance to the algorithm of the International Staging System (ISS). This score then additionally combined traditional ISS and LDH levels in a new score called revised-ISS, nowadays commonly used to stratify prognosis in newly diagnosed MM patients [38,80,81]. At diagnosis, a high risk disease based on genomic aberrations is defined by the presence of at least one between del(17p), t(4;14), t(14;16), with a median OS of 24.5 months. The absence, in a low ISS, defines standard risk with a median OS of 50.5 months [80]. Some international trials also define a category of patients with ultra-high risk basing on karyotype, who harbor more than one high risk lesion [82,83]. Table 2 defines risk stratifications of MM based on karyotype abnormalities used in most recent clinical trials.

Afterwards, other attempts to define prognostic scores were published. Perrot et al. proposed a cytogenetic prognostic score which divides patients in 3 categories based on 6 abnormalities—t(4;14), del(17p), trisomy 5, trisomy 21, 1q gain, del1p32 [84]. Interestingly, the first in literature, a recent WGS and WES study identified a subgroup of MM patients with good outcomes, independently by traditional clinical risk factor (R-ISS, treatment response and MRD negativity). It includes about 17% of newly diagnosed MM patients with lower mutational burden, few deletion events, and low genomic instability [85]. However, to date, these new prognostic tools are not widely applied in clinical trials. Table 3 and Table 4 present outcomes from diagnosis by genomic lesions in transplant eligible and ineligible MM patients, respectively, as reported in most recent studies. Overall, therapy intensification and continuous treatment seems to give significant benefit in high risk patients and in most of the genome aberrations. It is of particular interest that pembrolizumab did not show significant benefits in MM with complex genome aberrations, in contrast with results in other cancers [86,87]. Of note, t(11;14) has been identified as the first predictive marker of drug response to BCL2 inhibitors in MM, because of its association with high expression of BCL2 protein [88].

Table 5 shows a proposal of treatment recommendations based on cytogenetic risk that can be drawn out from randomized clinical trials. 

### 3.2. Drug-Resistance

MM is currently treated with a high number of drugs with distinctive mechanisms of action, used in combinations to optimize treatment and prognosis. The choice depends on age, comorbidities, and prognosis.

However, MM often shows occurrences of relapse and refractoriness, mostly due to drug resistance. Genetic instability and heterogeneity in MM are a main cause of drug resistance. Many studies evaluated the mechanisms of drug resistance in MM. Aberrations such as t(4;14), t(14;16), t(14;20), translocations involving c-MYC, 17p, and 13p deletions, have been associated with inadequate response to current treatments [89]. Translocation (4;14) results in overexpression of FGFR3 and MMSET carrying poor prognosis and resistance to alkylating agents but has been associated with survival benefit in patients treated with the proteasome inhibitor bortezomib [80,90]. Translocation (14;16) and (14;20) takes to overexpression of the MAF genes, leading to adverse clinical outcomes and drug resistance in some studies [91,92]. In patients with translocation t(4;14), the overexpression of FGFR3 takes to resistance to dexamethasone, but this is not the only one mechanism of drug resistance in MM cells carrying this abnormality [91,92]. Deletion 17p is associated with advanced stages disease, poor prognosis, and treatment failure due to dysregulation of TP53 ability to control cell cycle and apoptosis in response to DNA damage, as the same as TP53 gene mutations [93,94,95].

The progressive improvement of knowledge in MM biology will allow us to design specific drug combinations to overcome drug resistance. In last years, the use of new agents with specific targets is the best response to this therapeutic need.

### 3.3. Drug Targeting

Within the high number of mutated genes in MM, some of them could be potentially targeted by drugs. But this approach is limited by the presence of subclones harboring different gene mutations that could be paradoxically activated [10]. In order to overcome the effect of clonal heterogeneity and clonal selection, several new immunotherapeutic drugs have been developed, such as autoantibodies (anti-CD38, anti-CD319), chimeric antigen receptor (CAR-T) cells (against B-cell maturation antigen and Immunoglobulin K light chain), and immune-check point inhibitors, but their role in clonal progression needs to be further indagated [96,97,98,99,100,101].

CAR-T cell therapy is nowadays the most promising treatment against MM, under investigation in several clinical trials with preliminary controversial outcomes. Most outstanding results come from trials with CAR-T cells targeting BCMA [97]. Their extensive use is limited by some challenging side effects, i.e., cytokine release syndrome (CRS) and immune effector neurotoxicity syndrome (ICANS), and by their time consuming and expensive production and transportation requirements [102]. However, CAR-T cell therapy results in MM are overall promising, and this strategy needs to be improved.

## 4. Conclusions

We presented the tangle of genomic aberrations driving MM pathogenesis and progression, and we focused on their clinical impact. The last decade brings significant advantages in the biological knowledge of MM. Cheap and widely applicable sequencing technologies allow us to go through myeloma biology, and most recent clinical trials are taking advantages from preclinical data, building prognostic models that are also based on genome aberrations. To date, a divide between the enormous bulk of available preclinical data and the scalability of these data in the clinical setting still exists.

A better, genomic-based, prognostic model could have the capability to ameliorate patients’ outcomes. A major effort in incorporating a biology-driven approach in clinical trials is warranted. Genomic aberrations could serve to address precise therapy intervention other than for prognostication purposes. The description of clinical effect of genomic aberrations—at the clonal and sub-clonal level—and their impact on therapy response and clinical outcomes could help us in defining precise subset of patients to address with a certain treatment both at diagnosis and relapse, with the intent to create a personalized but evidence-based approach.

## Figures and Tables

**Table 1:** List of aberration deriving from genome or DNA damage in multiple myeloma, sorted by incidence or prevalence. Some digits may vary from original studies due to rounding.

Aberration	Incidence at Diagnosis	Prevalence	Citations
**Single nucleotide variants**			
MAPK pathway (mostly kRAS, nRAS, BRAF)		40%	[10,11,38]
NF-KB pathway		20%	[10,11,38]
DNA-repair pathway		15%	[38]
TP53 mut	2–4%	20–40%	[27,38,39,40,41]
**Chromosome-level events**			
1q gain	31–37%		[24,42,43]
+15	37%		[43]
+9	36%		[43]
+19	33%		[43]
+5	33%		[43]
+3	33%		[43]
+11	25%		[43]
+7	21%		[43]
6p gain	15%		[42,43]
+21	12%		[43]
11q gain	10%		[42,43]
+X	9%		[43]
13q loss	59–60%		[24,43]
16q loss	28–35%		[42,43]
1p loss	24–30%		[42,43]
14q loss	23–39%		[24,42,43]
8p loss	22–25%		[42,43]
6q loss	21–33%		[42,43]
−22	18%		[43]
16p loss	15%		[42]
12p loss	13–15%		[42,43]
−20	12%		[43]
del(17p)	7–10%	80%	[40,41,43]
**Hyperdyploidy**			
Hyperdyploidy	50%		[19,24,44,45,46]
**Translocations**			
t(11;14)(q13;q32) IGH/CCND1	15%		[19]
t(14;16)(q32;q23) IGH/MAF	5%		[19]
t(6;14)(p21;q32) IGH/CCND3	1–2%		[19]
t(14;20)(q32;q12) IGH/MAFB	1%		[19]
translocations involving MYC	15–20%		[19,47,48]
**Rare and complex events**			
Kataegis	3%		[19]
Chromothripsis	1–24%		[15,18,20]
Chromoanasynthesis	Anecdotical-19%	Anecdotical	[15,16,22]
Chromoplexy	4–11%	4%	[15,17,18]

**Table 2:** Risk stratification of MM based on karyotype abnormalities, as reported in recent clinical trials. IGH = immunoglobulin heavy chain, CCDN1 = cyclin D1, MAF = MAF BZIP transcription factor, MAFB = MAF BZIP transcription factor B.

Standard Risk	High Risk	Ultra-High Risk
Patients not in high risk or ultra-high risk group	t(11;14)(q13;q32) IGH/CCND1	More than 1 high risk lesion §
	t(14;16)(q32;q23) IGH/MAF	
	t(14;20)(q32;q12) IGH/MAFB	
	del (17p)	
	gain (1q) §	

**§** exclusively in some trials [82,83].

**Table 3:** Outcomes in transplant eligible patients with MM that were treated at diagnosis in large phase 3 trials according their genome aberrations. On 21 November 2020, we searched on PubMed (multiple myeloma AND phase 3 NOT relapse * NOT resist *) AND Clinical Trial[ptyp] AND “last 5 years”[PDat] and we received 39 results; these results were manually curated and all the trials including transplant eligible patients from diagnosis and presenting evaluable randomized data on prognostic impact of genomic aberrations in MM are included in this table. M = melphalan, V = bortezomib, P = prednisone, Dara = daratumumab, R = lenalidomide, D = dexamethasone, C = cyclophosphamide, T = talidomide, ASCT = autologous stem cell transplant, NA = not available, sCR = stringent complete response, CR = complete remission or better, MRD- = negative measurable residual disease, NR = not reached, CI = confidence interval, f-up = follow-up, * = statistically significant between study arms in sub-cohort analysis. Descriptive sentence or number of events with median follow-up are reported whenever median PFS and/or OS are not reported for the study. Some digit may vary from the original report due to rounding.

ID	Author (Year) Study Median f-up	Genomic Aberration	Treatment	Number of Patients with Genomic Aberration	MRD- n (%)	sCR, n (%)	CR, n (%)	ORR, n (%)	Median PFS (m)	Median OS (m)
1	F.M. Gay (2015) [103]	high risk cytogenetic as at least one abnormality: del(17p), t(4;14), or t(14;16)	CRD	30 (23%)	NA	NA	NA	NA	25 m (CI NA) *	NR
	median f-up 52 m		M200-ASCT	23 (18%)	NA	NA	NA	NA	33 m (CI NA) *	58 m (CI NA)
		t(4;14)	CRD	17 (13%)	NA	NA	NA	NA	NA	NA
			M200-ASCT	11 (9%)	NA	NA	NA	NA	NA	NA
		t(14;16)	CRD	6 (5%)	NA	NA	NA	NA	NA	NA
			M200-ASCT	6 (5%)	NA	NA	NA	NA	NA	NA
		del(17p)	CRD	10 (8%)	NA	NA	NA	NA	NA	NA
			M200-ASCT	6 (5%)	NA	NA	NA	NA	NA	NA
2	G. H. Jacskson (2019)—MYELOMA XI maintenance [82]	high risk as gain (1q), t(4;14), t(14;16), t(14, 20), del(17p)	R maintenance	102 (22%)	NA	NA	NA	NA	54 m (CI NA) *	NR
	median f-up 31 m		observation	56 (17%)	NA	NA	NA	NA	24 m (CI NA) *	NR
		ultra-high risk (more than one high risk lesions)	R maintenance	38 (8%)	NA	NA	NA	NA	23 m (CI NA) *	45 m (CI NA) *
			observation	23 (7%)	NA	NA	NA	NA	7 m (CI NA) *	33 m (CI NA) *
		t(4;14)	R maintenance	42 (9%)	NA	NA	NA	NA	18 events (43%) *	12 events (29%)
			observation	24 (7%)	NA	NA	NA	NA	24 events (100%) *	10 events (42%)
		t(14;16)	R maintenance	8 (2%)	NA	NA	NA	NA	4 events (50%)	3 events (37%)
			observation	6 (2%)	NA	NA	NA	NA	5 events (83%)	3 events (50%)
		del(17p)	R maintenance	25 (5%)	NA	NA	NA	NA	12 events (48%)	10 events (40%)
			observation	13 (4%)	NA	NA	NA	NA	10 events (77%)	7 events (54%)
		gain (1q)	R maintenance	104 (23%)	NA	NA	NA	NA	43 events (41%) *	24 events (23%)
			observation	58 (18%)	NA	NA	NA	NA	38 events (66%) *	17 events (29%)
3	G. H. Jacskson (2019)—MYELOMA XI intensification § [83]	high risk as gain (1q), t(4;14), t(14;16), t(14, 20), del(17p)	CVD intensification	32 (25%)	NA	NA	NA	NA	31 m (CI NA) *	9 events (28%)
	median f-up 30 m		no CVD intensification	48 (37%)	NA	NA	NA	NA	17 m (CI NA) *	11 events (23%)
		ultra-high risk (more than one high risk lesions)	CVD intensification	18 (14%)	NA	NA	NA	NA	24 m (CI NA) *	5 events (28%)
			no CVD intensification	12 (9%)	NA	NA	NA	NA	8 m (CI NA) *	7 events (58%)
		t(4;14)	CVD intensification	19 (7%)	NA	NA	NA	NA	12 events (63%) *	4 events (21%) *
			no CVD intensification	7 (2%)	NA	NA	NA	NA	7 events (70%) *	5 events (50%) *
		t(14;16)	CVD intensification	2 (1%)	NA	NA	NA	NA	2 events (100%)	1 event (50%)
			no CVD intensification	2 (1%)	NA	NA	NA	NA	2 events (100%)	1 event (50%)
		del(17p)	CVD intensification	5 (2%)	NA	NA	NA	NA	5 events (71%) *	2 events (29%) *
			no CVD intensification	12 (4%)	NA	NA	NA	NA	12 events (86%) *	8 events (57%) *
		gain (1q)	CVD intensification	25 (9%)	NA	NA	NA	NA	25 events (63%) *	12 events (30%)
			no CVD intensification	33 (11%)	NA	NA	NA	NA	33 events (72%) *	12 events (26%)
4	P. Moreau (2019)—CASSIOPEIA [104]	high risk as at least one abnormality: del(17p) or t(4;14)	Dara-VTD	82 (15%)	49 (60%)	20 (24%)	NA	NA	15 events (18%)	NA
	median f-up 18 m		VTD	86 (16%)	38 (44%)	24 (28%)	NA	NA	22 events (26%)	NA
5	M.A. Dimopoulos (2019)—TOURMALINE-MM3 [105]	high risk cytogenetic as at least one abnormality: del(17p), t(4;14), or t(14;16)	Ixazomib maintenance	61 (15%)	NA	NA	NA	NA	38 events (62%)	NA
	median f-up 31 m		placebo	54 (21%)	NA	NA	NA	NA	38 events (70%)	NA
6	M. Cavo (2020)—EMN02/H095 [106]	high risk as at least one abnormality: t(4;14), t(14;16), del(17p)	ASCT	135 (25%)	NA	NA	NA	NA	37.3 m (HR 0.63, 0.46–0.88) *	NR (HR 0.66, 0.45–0.99) *
	Median f-up 42 m		VMP intensification	90 (25%)	NA	NA	NA	NA	20.3 m (HR 0.63, 0.46–0.88) *	51 m (HR 0.66, 0.45–0.99) *
			single ASCT	42 (25%)	NA	NA	NA	NA	27 m (HR 0.59, 0.34–1.03)	54.7% (HR 0.70, 0.35–1.42)
			double ASCT	39 (22%)	NA	NA	NA	NA	46 m (HR 0.59, 0.34–1.03)	61.3% (HR 0.70, 0.35–1.42)
		del(17p)	ASCT	64 (11%)	NA	NA	NA	NA	NA	NR (HR 0.48, 0.27–0.86) *
			VMP intensification	41 (10%)	NA	NA	NA	NA	NA	47 m (HR 0.48, 0.27–0.86) *
			single ASCT	22 (13%)	NA	NA	NA	NA	30 m (HR 0.24, 0.09–0.66) *	57.1% (HR 0.30, 0.08–1.08)
			double ASCT	18 (10%)	NA	NA	NA	NA	NR (HR 0.24, 0.09–0.66) *	80.2% (HR 0.30, 0.08–1.08)
		t(4;14)	ASCT	63 (11%)	NA	NA	NA	NA	NA	NA
			VMP intensification	48 (12%)	NA	NA	NA	NA	NA	NA
			single ASCT	16 (9%)	NA	NA	NA	NA	NA	NA
			double ASCT	20 (11%)	NA	NA	NA	NA	NA	NA
		t(14;16)	ASCT	20 (4%)	NA	NA	NA	NA	NA	NA
			VMP intensification	15 (4%)	NA	NA	NA	NA	NA	NA
			single ASCT	7 (4%)	NA	NA	NA	NA	NA	NA
			double ASCT	6 (3%)	NA	NA	NA	NA	NA	NA

**§** regardless of transplant eligibility.

**Table 4:** Outcomes in transplant ineligible patients with MM that were treated at diagnosis in large phase 3 trials according to their genome aberrations. On 21 November 2020, we searched on PubMed (multiple myeloma AND phase 3 NOT relapse * NOT resist *) AND Clinical Trial[ptyp] AND “last 5 years”[PDat] and we received 39 results; these results were manually curated and all the trials including transplant ineligible patients from diagnosis and presenting evaluable randomized data on prognostic impact of genomic aberrations in MM are included in this table. M = melphalan, V = bortezomib, P = prednisone, Dara = daratumumab, R = lenalidomide, D = dexamethasone, Pembro = pembrolizumab, C = cyclophosphamide, T = talidomide, K = karfilzomib, NA = not available, sCR = stringent complete response, CR = complete remission or better, MRD- = negative measurable residual disease, NR = not reached, CI = confidence interval, f-up = follow-up, * = statistically significant between study arms in sub-cohort analysis. Descriptive sentence or number of events with median follow-up are reported whenever median PFS and/or OS are not reported for the study. Some digit may vary from the original report due to rounding.

ID	Author (Year) Study Median f-up	Genomic Aberration	Treatment	Number of Patients with Genomic Aberration	MRD- n (%)	sCR, n (%)	CR, n (%)	ORR, n (%)	Median PFS (m)	Median OS (m)
1	S. Zweegman (2016)—HOVON87/NMSG18 [107]	del (17p)	MPT-T	25 (7.9%)	NA	NA	NA	NA	20 events (80%)	11 events (44%)
	median f-up 36 m		MPR-R	19 (6%)	NA	NA	NA	NA	18 events (95%)	10 events (52%)
		t(4;14)	MPT-T	21 (6.6%)	NA	NA	NA	NA	20 events (95%)	12 events (57%) *
			MPR-R	19 (6%)	NA	NA	NA	NA	17 events (89%)	6 events (32%) *
		1q21 gain	MPT-T	64 (20.1%)	NA	NA	NA	NA	49 events (77%)	29 events (45%)
			MPR-R	67 (21.1%)	NA	NA	NA	NA	51 events (76%)	23 events (34%)
2	T. Facon (2018)—FIRST [108]	high risk cytogenetic as at least one abnormality: del(17p), t(4;14), or t(14;16)	RD continuous	43 (17%)	NA	NA	5 (12%)	33 (77%)	8.4	29.3
	median f-up 67 m		RD18	52 (20%)	NA	NA	7 (13%)	35 (67%)	17.5	24.3
			MPT	47 (19%)	NA	NA	0	32 (68%)	14.6	35.5
3	M.V. Mateos (2018), M.V. Mateos (2019)—ALCYONE [109,110]	high risk cytogenetic as at least one abnormality: del(17p), t(4;14), or t(14;16)	Dara-VMP	53 (14.9%)	NA	10 (19%)	22 (42%) *	49 (92.5%) *	NA	NR
	median f-up 40 m		VMP	45 (12.9%)	NA	1 (2%)	11 (24%) *	33 (73.3%) *	NA	39.2
4	T. Facon (2019)—CLARION [111]	high risk cytogenetic as at least one abnormality: del(17p), t(4;14), or t(14;16)	KMP	54 (11.3%)	NA	NA	NA	NA	ns between arms; HR 0.96 (95% CI: 0.58–1.58)	NA
	median f-up 27 m		VMP	67 (14%)	NA	NA	NA	NA		NA
5	G. H. Jacskson (2019)—MYELOMA XI maintenance [82]	high risk as gain (1q), t(4;14), t(14;16), t(14, 20), del(17p)	R maintenance	64 (27%)	NA	NA	NA	NA	16 m (CI NA) *	49 m (CI NA)
	median f-up 31 m		observation	57 (28%)	NA	NA	NA	NA	8 m (CI NA) *	46 m (CI NA)
		ultra-high risk (more than one high risk lesions)	R maintenance	15 (6%)	NA	NA	NA	NA	14 m (CI NA)	46 m (CI NA)
			observation	7 (3%)	NA	NA	NA	NA	11 m (CI NA)	39 m (CI NA)
		t(4;14)	R maintenance	9 (4%)	NA	NA	NA	NA	7 events (78%)	3 events (34%)
			observation	8 (4%)	NA	NA	NA	NA	8 events (100%)	5 events (62%)
		t(14;16)	R maintenance	13 (5%)	NA	NA	NA	NA	11 events (85%)	8 events (62%)
			observation	2 (1%)	NA	NA	NA	NA	1 event (50%)	1 event (50%)
		del(17p)	R maintenance	12 (5%)	NA	NA	NA	NA	8 events (67%)	5 events (42%)
			observation	11 (5%)	NA	NA	NA	NA	11 events (100%)	7 events (64%)
		gain (1q)	R maintenance	58 (24)	NA	NA	NA	NA	60 events (62%) *	27 events (47%)
			observation	49 (24%)	NA	NA	NA	NA	46 events (94%) *	23 events (47%)
6	S.Z. Usmani (2019)—KEYNOTE-185 [100]	high risk cytogenetic as at least one abnormality: del(17p), t(4;14), or t(14;16)	Pembro-RD	24 (16%)	NA	NA	NA	NA	NA	4 events (21%)
	median f-up: 6.6 m		RD	10 (7%)	NA	NA	NA	NA	NA	0 events
7	P.M. Voorhees (2020)—GRIFFIN [112]	high risk cytogenetic as at least one abnormality: del(17p), t(4;14), or t(14;16)	RVd	14 (14.4%)	53 (51%)	42 (42.4%)	9 (9.1%)	98 (99%)	NR	NR
	median f-up 22 m		Dara-RVd	16 (16.3%)	21 (20.4%)	31 (32%)	10 (10.3%)	89 (91.8%)	NR	NR
8	S.K. Kumar (2020)—ENDURANCE [113]	t(4;14)	VRd	44 (12%)	NA	NA	NA	NA	18.3 m (14.9-NR)	NA
	median f-up 9 m		KRd	36 (9%)	NA	NA	NA	NA	19.3 m (12.7-NR)	NA

**Table 5:** Recommendations of cytogenetic-driven therapy in newly diagnosed multiple myeloma patients. V = bortezomib, D = dexamethasone, C = cyclophosphamide, ASCT = autologous stem cell transplant.

Patient Population	Recommendations	Evidences for Recommendations
Transplant eligible patients	No clear advantage has been demonstrated in adding daratumumab in first line therapy to bortezomib-based regimens in high risk patients.	Median PFS 26% VTD vs. 18% Dara-VTD [104]
	CVD intensification after induction therapy should be offered to high and ultra-high cytogenetic risk patients, including patients with gain 1q and t(14;20), not achieving at least a VGPR before proceeding to ASCT.	Median PFS 31 months with CVD intensification vs. 17 months without CVD intensification [83]
	Melphalan 200 mg/sqm followed by ASCT should be preferred to consolidation with CRD in high cytogenetic risk patients.	Median PFS 25 months CRD vs. 33 months M200-ASCT [103]
	ASCT should be offered in high cytogenetic risk patients, particularly in del(17p). Furthermore, double ASCT should be preferred to single ASCT in del(17p).	Median PFS 37 m with ASCT vs. 20 m with VMP (HR 0.63, 0.46–0.88). Median OS not reached with ASCT vs. 52 m with VMP (HR 0.66, 0.45–0.99). Median PFS not reached with double ASCT vs. 30 m with single ASCT in del(17p) patients (HR 0.24, 0.09–0.66) [106]
	Lenalidomide maintenance should be offered to high and ultra-high cytogenetic risk patients, including patients with gain (1q) and t(14;20).	Median PFS 54 months with R maintenance vs. 24 months with observation) [82]
Transplant ineligible patients	Lenalidomide based-regimen should be preferred in t(4;14) patients.	Better OS; 6 deaths with MPR-R vs. 12 deaths with MPT-T [107]
	Daratumumab should be added in first line therapy to bortezomib-based triplets in patients with high cytogenetic risk.	ORR 92.5% in Dara-VMP vs. 73.3% in VMP [109,110]
	Lenalidomide maintenance should be offered to high cytogenetic risk patients, including patients with gain (1q) and t(14;20), but benefit is not clear in ultra-high risk patients.	Median PFS 16 months with R maintenance vs. 8 months with observation [82]
